# In Nature's School

**Published:** 1889-08-03

**Authors:** 


### The Doctor's Pulpit.

### IN NATURE'S SCHOOL.

### Beauty and Use.

The forms and colours which Nature displays for the admiration of man are admittedly beautiful. Very few painters have ventured to ignore Nature: none have dared to flagrantly contradict her; because they have known that such contradiction would inflict irreparable damage on their fame. It is true that milliners and dressmakers have often outraged Nature in the most daring and absurd ways. But whoever thinks of calling a milliner or a dressmaker an artist? In spite of the recent prevalence of neutral tints in what is called "high art," nobody has yet suggested that the primary colours are ugly. The scarlet poppies in the corn-field are still the painter's delight, no matter how "glaring" the scarlet may be. The green of the grass or of the young oak leaves can never be too universal or too bright, though it may be esteemed vulgar by the disciples of Worth. The blue of the cloudless midsummer sky would be held intolerable for many purposes; but what painter would not give five years of his lifetime if he could reproduce on his canvas the glorious azure that envelopes the world? Similarly, the forms of Nature are models which he whose genius is greatest always most longs to imitate. The noble and massive trunks of the forest tree; the spreading branches of the venerable oak; the lithe, willowy grace of the sapling; the incomparable sepals, petals, stamens, and pistils of the flowers; to say nothing of the limbs of the four-footed creatures, the wings and other delicate structures of insect life—these all, for variety, grace, delicacy, and perfectness, are beyond admiration.

On this all are agreed; the creationists and evolutionists are entirely at one. When we come to consider the further question of use as well as beauty, sharp contrasts of opinion at once become evident. Is everything in Nature useful as well as beautiful? Many who admit that nearly everything is beautiful, will deny that everything is designed to serve a useful purpose. Perhaps no thinking man would care to show himself so inexperienced as to affirm that every created thing was originally made for some definite, foreseen, and pre-arranged use. It may be even so. But there is no necessity for any writer who has a practical purpose to serve to commit himself to a universal proposition of that kind. What is manifest to every clear-eyed and candid person is this, that practically all things in Nature are useful as well as beautiful, or may be made so. In other words, Nature believes in results; she constantly produces results; purposeless and resultless work is not to her mind. If a corn of wheat fall into the ground it germinates, and in the course of a few months brings forth a hundred other corns of wheat of like size, shape, and character. If a pip be planted it strikes root into the soil and springs up into a tree. The tree produces branches, then leaves, then flowers, and last of all fruit. There is no way of proving that the tree actually grows for the purpose of producing fruit; but it is plain to the most ignorant and unconcerned observer that the last term of a series of developments is fruit; and that when the fruit has been yielded, a time of rest is entered upon, after which the whole series begins afresh, follows the same order, and closes with fruit as before. All sorts of series in Nature may appear to be endless, but at a definite point in each revolution of the wheel a fruit stage is reached. Not only is this the case with plants and trees, but the animal world is strictly analogous in all its details. Use, in fact, is in Nature as universal as beauty for all practical purposes. The whole world, in all its parts, is made for fruit and use, and only in fruit and use do living organisms find their highest development and perfection.

The world consists of man and all other living forms. Although man is above and beyond all other living forms, he is still in a large sense merely a step beyond the highest of the grades that are below him. He is so nearly related to other organic forms that what is predicable of them is to a large extent predicable also of him. Nature makes it quite plain that living organisms other than man are made for results; she makes it equally clear by analogy, as well as in other ways, that man, too, is intended to produce results. What is the most general law that determines the results which living forms ordinarily produce? The scientific law is that each organism shall produce fruit "after its kind." Exactly is this the case with man. He is intended by Nature to produce fruit "after his kind." And only in producing fruit after his kind can he find his highest perfection and development; only thus can he attain to a normal standard of health. For in man health is the ultimate test of constitutional soundness and right conduct.

"All roads lead to Rome," it used to be said, when Rome was mistress of the world. Similarly, it may be said that all the doings of man lead to health, or to its opposite. They ought to lead to health. Health in its largest sense is the grand test of the value of human ways, and human discoveries, and human progress. Does the life of a city, the discovery of a chemist, or the new religion of an enthusiast make for the *mens sana in sano corpore?* That is the final test by which it must be tried. Not beauty alone but beauty and use are Nature's rule. So determined is Nature that man shall be always and everywhere a useful creature that she will not grant him health on any other condition. His limbs, his brain, his stomach, his liver were all made for daily and effectual work. Deprive any or all of them for a time of their daily work and health will infallibly disappear. There are many other conditions necessary to perfect health as well as that of the energetic use of the parts of the body; but if every other condition, down to the minutest, be fulfilled exactly, there can still be no sound health without work both of body and brain. The muscles of the arms, trunk, and legs demand work, not only for their development and strengthening, but also that they may convert into waste that which waits to be so converted, and may then promptly get rid of it by the ordinary methods of physiology. Just as too much "bush" and "scrub" encumber the ground in primeval forests and make cultivation impossible until they are cleared away, so do the tissues of the body, if not converted into waste and got rid of with sufficient rapidity, place health and vigour entirely out of reach. Recently there have been certain protests made against the teaching of what we have called "Nature's School." Such protests can never make facts change their colour or natural processes their behaviour. Nature insists upon being obeyed to the letter; and every single act of disobedience receives, in the impressive language of holy Scripture, a "just recompense of reward."

Thousands of people of all classes are of no use whatever in the world; they have legs, but they never walk if they can ride; they have arms, but they cannot dig or even lift a boiling kettle from the fire. They have brains; but they can no more think an original and independent thought than Punch's dog Toby can bark and bite. They are mere waste and rubbish in the world's great farm, fit only to be gathered together into heaps like thorns and couch-grass, and burnt into white ash. Work—productive work—is the one thing in all the world on which Nature sets the sign and seal of her most perfect approval. What is the law of the survival of the fittest—a law which seems brutal when misunderstood, and which has afforded hard and selfish men a scientific excuse for some of the basest deeds the world has ever witnessed—what is that law but the voice of Nature, proclaiming to all who have ears that use and service are the highest products of every kind of activity? The fruit of the tree is now, as ever, the final test of its value.

